# Evaluation of occlusal status of Japanese adults based on functional tooth units

**DOI:** 10.1016/j.identj.2021.02.005

**Published:** 2021-05-06

**Authors:** Takashi Zaitsu, Mari Ohnuki, Yuichi Ando, Yoko Kawaguchi

**Affiliations:** a Department of Oral Health Promotion, Graduate School of Medical and Dental Sciences, Tokyo Medical and Dental University, Tokyo, Japan; b Department of Health Promotion, National Institute of Public Health, Saitama, Japan

**Keywords:** Dental prostheses, National health survey, Tooth loss, Occlusal status, Japanese adults

## Abstract

**Objectives:**

Although extensive national oral health data on dental caries and periodontal diseases in Japan are available, few studies have assessed the occlusal status of the Japanese population, and none are based on national survey data. The presence and prosthodontic conditions of the molar region are important for masticatory function, and the functional tooth unit (FTU) approach can be used to evaluate the occlusal status. Thus, using the national oral health survey data, this study investigated the occlusal status of the Japanese population using FTU.

**Methods:**

Overall, 3,605 adults (aged ≥20 years) who participated in the 2011 Japanese national oral health survey were included. FTUs were used as indices for evaluating the occlusal status. FTUs were calculated according to sex, age group, and the number of teeth present, and their associations were further analysed.

**Results:**

The number of teeth present, posterior teeth, and FTUs decreased with age in both men and women. In the age group of those ≥60 years, all only natural teeth-FTU (n-FTU) and natural teeth and artificial teeth from fixed prostheses or implant-supported FTU (nif-FTU) scores were <8. The total-FTU scores of all age groups, except the 60-69 and 70-79 years age groups, were >10.

**Conclusion:**

This is the first study to use FTUs and national oral health survey data to investigate the occlusal status in the Japanese population. People aged ≥60 years who have low n-FTU or natural teeth and artificial teeth from fixed prostheses or implant-supported FTU scores or those aged 60-70 years who have the lowest total-FTU scores require careful evaluation of masticatory performance.

## Introduction

The national oral health survey has been conducted every 5-6 years in Japan since 1957.^1^ These surveys report the dental, prosthetic, and gingival status and malocclusion in all age groups in the Japanese population. Thus, many studies on dental caries and periodontal diseases in the Japanese population have been conducted based on these reports.^2^, ^3^, ^4^ The results of these surveys have suggested that many individuals in older age groups have missing teeth and a higher rate of use of prostheses. However, these studies have not investigated the occlusal status, which is strongly associated with masticatory ability.

Many indices, such as the Kennedy classification and Eichner index, are used to evaluate occlusal status.^5^, ^6^, ^7^, ^8^, ^9^, ^10^, ^11^ These indices are useful clinical classifications for revealing the exact prosthetic requirements and design of dentures for individual patients. However, the occlusal status of molars cannot be easily summarised with these indices. Hence, it is difficult to use them for the classification of prosthetic treatment needs or chewing function in a community.

The functional tooth unit (FTU) is an index that defines pairs of opposing teeth.^12^, ^13^, ^14^ The occlusal status can be easily computed using FTUs based on the presence or absence of molars and premolars. Previous studies have reported that the FTU is associated with nutrition and various health problems. In particular, the risk of malnutrition is higher in elderly people with low FTU scores and people requiring long-term care,^14^^,^^15^ and low FTU scores affect health conditions such as obesity in Japanese people.^16^ However, few studies have investigated the occlusal status of the Japanese population using national survey data, despite the importance of evaluating the occlusal status and considering countermeasures. Thus far, in Japan, the number of teeth, periodontal disease, and dental caries have been investigated according to age, and the specific target in each age group has been set in Healthy Japan 21. In Healthy Japan 21, the goals of nutrition and obesity, which are strongly related to the occlusal condition, were set. However, the clinical occlusal condition of the Japanese population has not been investigated by chronological age and sex, and the problem has not been addressed. In Japan, FTU scores have been used to evaluate the oral function and masticatory efficiency of a community of people in a specific area.^17^^,^^18^ In those studies, lower masticatory function has been reported in individuals with all only natural teeth-FTU (n-FTU) and natural teeth and artificial teeth from fixed prostheses or implant-supported FTU (nif-FTU) scores <8 or total-FTU scores <10. In addition, evaluating the occlusal condition by age group is important to understand the problems of the entire population. According to previous studies, FTU scores decrease in individuals aged 50-59 years.^13^ It is important to investigate the occlusal status by age and sex in the entire Japanese population to understand the problem because it often shows different patterns for specific areas as well as the entire country.

This study aimed to clarify the occlusal status, which has not been previously investigated in Japan, by evaluating the occlusal status using FTU in the Japanese population according to sex and chronological age. This study provides a more complete picture of the oral health needs of the Japanese population and analyse their potential prosthodontic treatment requirements, making it possible to provide basic data that can be compared internationally with regard to future national goals.

## Methods

### Subjects

We included Japanese residents who participated in the Japanese national oral health survey in 2011.^1^ Some aspects of oral health status in Japan have been assessed at the national level using reports from the Survey of Dental Diseases (SDD). SDD is conducted every 5-6 years since 1957. We obtained SDD data for 2011 from the Ministry of Health, Labour and Welfare with permission. SDD uses a hierarchized 2-stage cluster sample design to obtain a nationally representative sample of the noninstitutionalised Japanese population. The sampling frame is a list of the enumeration areas of all residential censuses stratified into 47 prefectures. Each population survey area consisted of approximately 50 households. Ultimately, 150-unit blocks were extracted from each prefecture.^19^ These data are for Japanese people aged at least 1 year; the data set in 2011 comprised 4253 people (1812 men and 2441 women). For this study, we included subjects aged ≥20 years. Our analysis, therefore, included 3605 adults (1485 men and 2120 women), with a mean age of 58.3 ± 17.0 years.

For this study, informed consent was not required, and ethical approval was not necessary as the data for this study were obtained from national surveys as per the Japanese ethical guidelines for medical and health research involving human subjects.^20^

### Parameters investigated

For the national survey, the dental examination was conducted with a dental mirror and a World Health Organization (WHO)-type periodontal probe. Dental status was examined visually and by tactile inspection.^21^ All examiners received calibration training using the criteria manual based on the WHO oral health examination methods.^21^

For this study, data on demographic and clinical characteristics including sex, age, and the condition of each tooth were obtained from the national oral survey conducted in November 2011. Patients were divided into different age groups (20-29, 30-39, 40-49, 50-59, 60-69, 70-79, and ≥80 years). The number of teeth present, posterior teeth, and FTUs were calculated based on the original dental examination chart, excluding the third molars.

The FTUs were defined as pairs of opposing natural teeth (sound teeth, restored teeth, and decayed teeth with preserved tooth crown), artificial teeth that were fixed (bridge pontics) or implant supported, and removable prostheses. Missing teeth and decayed teeth with roots were defined as not functional. Two premolars that opposed each other were defined as 1 FTU, and 2 molars that opposed each other were defined as 2 FTUs. Therefore, for a person with a complete dentition (third molars excluded), the highest attainable score was 12 FTUs. Based on tooth composition, the FTUs were classified as n-FTUs, nif-FTUs, and complete FTUs (natural teeth, artificial teeth, and removable prostheses: total-FTUs).

### Statistical analysis

One-way analysis of variance was used to detect any significant differences in the mean number of total teeth present, posterior teeth, and FTUs (n-FTUs, nif-FTUs, and total-FTUs) between sexes and among the different age groups. The Jonckheere–Terpstra test was performed to determine whether a particular trend was present. A *P* value of <.05 was considered statistically significant. SPSS 20.0J software package (SPSS Japan) was used for analysis.

## Results

### Number of teeth present and number of posterior teeth by sex and age group

Table 1 shows the mean number of posterior teeth and total teeth by sex and age group. Men had a higher overall number of teeth present and had more posterior teeth than women in the 20-29 and ≥80 age groups. However, no difference was observed between men and women in the other age groups. In both men and women, the number of posterior teeth and total teeth was greater in the younger age groups than in the older age groups (*P* value for trend <.001).

**Table 1:** Mean number of total and posterior teeth by age and sex.

Age group	Total			Men			Women			*P* value
(years)	*N*	Mean	SD	*n*	Mean	SD	*n*	Mean	SD	
Present teeth										
20-29	211	27.52	1.24	77	27.71	0.89	134	27.40	1.39	**.048**
30-39	464	27.34	1.29	177	27.32	1.28	287	27.36	1.29	.730
40-49	437	26.64	3.00	157	26.43	3.64	280	26.76	2.58	.265
50-59	543	24.48	4.83	213	24.14	5.12	330	24.69	4.62	.195
60-69	835	21.46	7.13	354	21.42	7.47	481	21.50	6.87	.871
70-79	784	16.26	9.47	365	16.17	9.75	419	16.35	9.23	.793
≥80	331	10.72	9.43	142	12.00	9.59	189	9.76	9.21	**.032**
*P* value for trend	*P* < .001			*P* < .001			*P* <.001			
Posterior teeth										
20-29	211	15.59	1.10	77	15.78	0.87	134	15.49	1.20	**.041**
30-39	464	15.42	1.20	177	15.38	1.23	287	15.45	1.18	.558
40-49	437	14.93	2.11	157	14.89	2.33	280	14.95	1.99	.746
50-59	543	13.19	3.53	213	12.97	3.73	330	13.34	3.40	.231
60-69	835	11.12	4.53	354	11.19	4.58	481	11.08	4.50	.735
70-79	784	8.20	5.45	365	8.34	5.56	419	8.07	5.36	.497
≥80	331	5.08	5.09	142	5.88	5.22	189	4.48	4.91	**.013**
*P* value for trend	*P* < .001			*P* < .001			*P* < .001			

SD = standard deviation.

### FTU score (n-FTUs, nif-FTUs, and total-FTUs) by sex and age group

Table 2 shows the mean FTU scores (n-FTUs, nif-FTUs, and total-FTUs) by sex and age group. All 3 types of FTUs were higher in the younger age groups than in the older age groups in both sexes (*P* value for trend <.001).

**Table 2:** Mean number of different types of FTUs by age and sex.

Age group	Total			Men			Women			*P* value
	*N*	Mean	SD	*n*	Mean	SD	*n*	Mean	SD	
n-FTUs										
20-29	211	11.57	1.16	77	11.79	0.69	134	11.45	1.34	**.015**
30-39	464	11.25	1.58	177	11.19	1.63	287	11.28	1.55	.566
40-49	437	10.59	2.47	157	10.45	2.72	280	10.66	2.31	.397
50-59	543	8.34	3.82	213	8.13	3.92	330	8.48	3.75	.299
60-69	835	6.07	4.25	354	6.08	4.20	481	6.06	4.29	.968
70-79	784	3.91	4.24	365	4.03	4.33	419	3.82	4.17	.487
≥80	331	1.88	3.24	142	2.27	3.51	189	1.59	3.00	.062
*P value* for trend	*P* < .001			*P* < .001			*P* < .001			
nif-FTUs										
20-29	211	11.63	1.09	77	11.86	0.58	134	11.49	1.28	**.005**
30-39	464	11.41	1.29	177	11.33	1.44	287	11.46	1.20	.307
40-49	437	10.98	2.22	157	10.89	2.47	280	11.04	2.07	.488
50-59	543	9.36	3.70	213	9.17	3.90	330	9.49	3.57	.335
60-69	835	7.24	4.55	354	7.24	4.54	481	7.24	4.56	.990
70-79	784	4.64	4.71	365	4.84	4.79	419	4.45	4.64	.247
80	331	2.24	3.75	142	2.67	3.93	189	1.92	3.58	.076
*P* value for trend	p<0.001			p<0.001			p<0.001			
Total-FTUs										
20-29	211	11.63	1.09	77	11.86	0.58	134	11.49	1.28	**.005**
30-39	464	11.45	1.20	177	11.41	1.27	287	11.48	1.16	.501
40-49	437	11.15	1.85	157	11.08	2.12	280	11.18	1.68	.591
50-59	543	10.28	2.78	213	10.01	3.13	330	10.46	2.51	.066
60-69	835	9.83	3.06	354	9.76	3.21	481	9.88	2.95	.573
70-79	784	9.98	2.98	365	10.17	2.84	419	9.82	3.09	.100
≥80	331	10.16	3.23	142	10.24	2.94	189	10.10	3.43	.699
*P* value for trend	*P* < .001			*P* < .001			*P* < .001			

FTUs = functional tooth units; n-FTUs = natural teeth FTUs; nif-FTUs =natural teeth and artificial teeth of fixed prostheses or implant-supported FTUs; SD = standard deviation.

In the 20- to 29-years age group, men had significantly higher FTU scores (n-FTUs, nif-FTUs, and total-FTUs) than women. However, no significant differences were observed between men and women in the other age groups. In the ≥60 years age groups, all n-FTU and nif-FTU scores were <8, and the total-FTU scores were >10 in all age groups, except in the 60- to 79-years age group.

## Discussion

This is the first study to use FTUs and national oral health survey data to investigate the occlusal status of the Japanese population. We observed that the number of natural teeth present, n-FTU scores, and nif-FTU scores were lower in the older age groups than in the younger age groups.

FTUs can be easily digitised using simple calculations of posterior tooth occlusion. The FTU is a useful tool that can be used to evaluate the occlusal status in a large population. A previous study reported that a lower number of FTUs was associated with chewing difficulties and poor oral function.^15^ It revealed that maintaining an n-FTU or nif-FTU score of at least 8 or a total-FTU score of at least 10 is important in the prevention of the development of chewing difficulties.

The present study showed that the number of teeth present, number of posterior teeth, n-FTU scores, and nif-FTU scores had the highest values in the 20- to 29-years age group and the lowest values in the ≥80 years age group. These scores decreased with age in both men and women. In particular, in the ≥60 years age groups, both n-FTUs and nif-FTU scores were >8. However, the lowest total-FTU scores were in the 60- to 69- and 70- to 79-years age groups (score <10).

The results of this study indicate that elderly people had greater tooth loss and that their missing teeth were replaced with removable prostheses. This may be attributed to the Japanese public health insurance system. In Japan, the universal health insurance system for the entire population was established in 1961. It covers almost all medical and dental treatments as well as any pharmacy care needed by the population.^22^^,^^23^ People can receive restorative, prosthetic, and oral surgery treatment at a relatively low cost, and the same fee is applied throughout the nation.^24^^,^^25^ This system allows elderly people who have lost their natural teeth to receive prosthetic treatment, and their functional occlusal status appears to be better than that of people in other countries. Compared with elderly people in other countries, elderly Japanese people in this study had a higher number of teeth and FTU scores.^14^^,^^26^^,^^27^

However, despite such a positive situation in Japan, the n-FTU and nif-FTU scores in the ≥60 years age groups were <8. In addition, the total-FTU in the 60- to 79-years age group was <10. The SSD data suggest the possibility that as chewing difficulties increase with an increasing number of missing teeth, this increases the likelihood of seeking prosthetic treatment.^1^ Furthermore, with the increasing possibility of undergoing prosthetic treatment after tooth loss, the likelihood of undergoing treatment with removable prostheses with dentures (not a fixed prosthesis, such as an implant or a bridge) increases. Moreover, women in their 20s had a lower total number of teeth present, number of posterior teeth, n-FTU scores, nif-FTUs scores, and total-FTU scores than men of the same age. Women often undergo tooth extraction in the premolar region for orthodontic reasons.^28^ In another study, women in their 20s had more tooth extractions than men and 4 times more orthodontic tooth extractions than men.^29^ However, the number of males (211 people) in this study was small, and the standard deviation was also small. It was also possible that younger men with particularly good oral conditions were more likely to have participated in this study. In the ≥80 years age group, women had a lower number of teeth than men, but no significant difference in the FTU scores was observed. The standard deviation of the mean values of the present teeth in elderly people was large, suggesting that there may be considerable variation in the presence of teeth in elderly people. However, there were few differences in total-FTU scores between the prosthetic procedures performed. There is the possibility that older Japanese women receive prosthetic treatment and maintain occlusion better than men with a large number of missing teeth.^30^^,^^31^

There is a limitation to the evaluation of occlusal status in this study. The FTU index used in this study was based only on the presence or absence of maxillary-mandibular molar and premolar pairs, and this does not indicate the clinical diagnosis of the presence or absence of direct occlusal contact. The FTU is used for the self-assessment of the occlusion status, and it is an index of masticatory ability.^15^^,^^16^ Therefore, in epidemiological surveys of a large number of people, the FTU is a useful index to assess the chewing ability and occlusion status. It is important to appropriately use both the FTU and other classifications of occlusion, such as the Kennedy classification and Eichner index, to evaluate the clinical occlusion status and prosthetic needs of each patient. Moreover, further research is needed to investigate the association between FTUs and other occlusal evaluations in the Japanese population. There is also a limitation in the sample size. The number of subjects in this study was relatively small (*n* = 3605), and there is a concern that they may not sufficiently represent the Japanese population. However, as described in the Methods section, the approach to selecting samples was precise, and hence, the included participants can be considered to represent the entire Japanese population. In addition, this study is a cross-sectional survey, and a comparison of occlusal status in longitudinal studies is necessary. In addition, inadequate clinical calibration for the examiner could be cited as a limitation. There were no calibration data or intrareliability or inter-reliability information. However, the study was performed using a strict manual based on the WHO oral health examination methods.^21^ In addition, biases in health outcomes due to inadequate calibration are considered to result in nondifferential misclassification.

## Conclusion

This study revealed the detailed characteristics of the occlusal status of the Japanese population. Japanese people maintained good occlusal status, even during old age (≥80 years) by replacing missing teeth with removable prostheses. However, people aged ≥60 years who have low n-FTU or nif-FTU scores or people in their 60s-70s who have the lowest total-FTU scores require careful masticatory evaluation.

The findings of this study should help in the planning of future oral health measures and the development of guidelines for oral care as the needs of the Japanese population change. In Healthy Japan 21, the Ministry of Health, Labour and Welfare of Japan has included improved goals for dental caries, periodontal disease, and the number of teeth for the Japanese people. Data on the occlusal status of the Japanese population are scarce. Therefore, in the future it is important to incorporate the occlusal status using FTUs into future national dental surveys.

## Funding

This study was supported by the Research Fund of Clinical Study for Industrial Accident and Disease of the Ministry of Health, Labor, and Welfare, Japan (170501-01).

## Conflict of interest

The authors declare that they have no conflicts of interest.

## Appendix Supplementary materials

Appendix Fig A. Occlusal status of opposing tooth pairs by age group (%) Image, application 1
