# Supplementary material for: Evaluation of occlusal status of Japanese adults based on functional tooth units
Source: Int Dent J. 2021 May 6;72(1):100–5. doi: 10.1016/j.identj.2021.02.005 (PMC9275336; doi:10.1016/j.identj.2021.02.005)
Supplement: Supplementary file 1 — Appendix Fig A. Occlusal status of opposing tooth pairs by age group (%) [file mmc1.docx]

Fig. A The occlusal status of opposing tooth pairs in different age groups (%)

Opposing tooth pairs comprise

n–n: natural to natural teeth,

f–n: fixed prosthetic to natural teeth,

f–f: fixed to fixed prosthetic teeth,

r–n: removable prosthetic to natural teeth,

r–f: removable to fixed prosthetic teeth,

r–r: removable to removable prosthetic teeth,
